# Force enhancement after stretch of isolated myofibrils is increased by sarcomere length non-uniformities

**DOI:** 10.1038/s41598-020-78457-1

**Published:** 2020-12-09

**Authors:** Ricarda M. Haeger, Dilson E. Rassier

**Affiliations:** Kinesiology and Physical Education, McGill University, Montreal, QC Canada

**Keywords:** Biophysics, Physiology

## Abstract

When a muscle is stretched during a contraction, the resulting steady-state force is higher than the isometric force produced at a comparable sarcomere length. This phenomenon, also referred to as residual force enhancement, cannot be readily explained by the force-sarcomere length relation. One of the most accepted mechanisms for the residual force enhancement is the development of sarcomere length non-uniformities after an active stretch. The aim of this study was to directly investigate the effect of non-uniformities on the force-producing capabilities of isolated myofibrils after they are actively stretched. We evaluated the effect of depleting a single A-band on sarcomere length non-uniformity and residual force enhancement. We observed that sarcomere length non-uniformity was effectively increased following A-band depletion. Furthermore, isometric forces decreased, while the percent residual force enhancement increased compared to intact myofibrils (5% vs. 20%). We conclude that sarcomere length non-uniformities are partially responsible for the enhanced force production after stretch.

## Introduction

The amount of force produced by a muscle fiber is dictated by its length, giving rise to the well-known force–length relation. This relation was established several decades ago by a classic study using isolated muscle fibers^1^ and has been repeated in several laboratories^2–4^. It shows that, under isometric conditions, the force should be proportional to the degree of overlap between myosin and actin filaments. However, when a muscle fiber is stretched *while* activated, the steady-state force stabilizes at a level that is higher than that produced during isometric contractions at the corresponding length^5–8^. The mechanism behind this phenomenon, also known as residual force enhancement, remains elusive as it cannot be explained by the force–length relation^9,10^. Residual force enhancement is present in muscle fibers^5–7^, myofibrils^11,12^, and single sarcomeres^8^, with increases in force of up to ~ 28%.

One of the most accepted explanations for residual force enhancement is the development of sarcomere length non-uniformities within a myofibril during activation and after stretch^6,13,14^. Stretching increases the amount of the already existent inhomogeneity of sarcomere lengths: weak, long sarcomeres will be stretched, and stronger sarcomeres will shorten^7,8^. Accordingly, the strong sarcomeres will have a filament overlap larger than the average sarcomere length, thus producing more force than expected. The long sarcomeres will equilibrate the total force by increasing the passive force. In this way, an increase in sarcomere length non-uniformity beyond levels that are present during isometric contractions will lead to an increased total force produced after stretch. Furthermore, in a recent study performed in our laboratory, we observed that force enhancement was directly linked to the non-uniformity of *half*-sarcomeres; when half-sarcomere length non-uniformity was not present, the levels of force enhancement were substantially decreased or even inexistent^8^. This potential mechanism for residual force enhancement has not been without controversy^8,15^, and the role of sarcomere length non-uniformities needs further clarification.

In this study, we investigated the effect of sarcomere length non-uniformities on the residual force enhancement by inducing sarcomere length non-uniformities in isolated myofibrils—preparations in which we can track all sarcomeres during activation and stretch. We developed a new technique in our laboratory that can target selected sarcomeres to be treated with a high ionic strength solution^16,17^, causing a depletion of sarcomere thick filaments. Such a procedure leads to a lack of inter-sarcomere communication in myofibrils^16,17^. The sarcomere lacking the thick filament becomes weak and will elongate, increasing the sarcomere length non-uniformity in myofibrils. We hypothesized that the increase in sarcomere length non-uniformity would lead to increased levels of residual force enhancement.

## Results

### A-Band extraction

**Figure 1:**
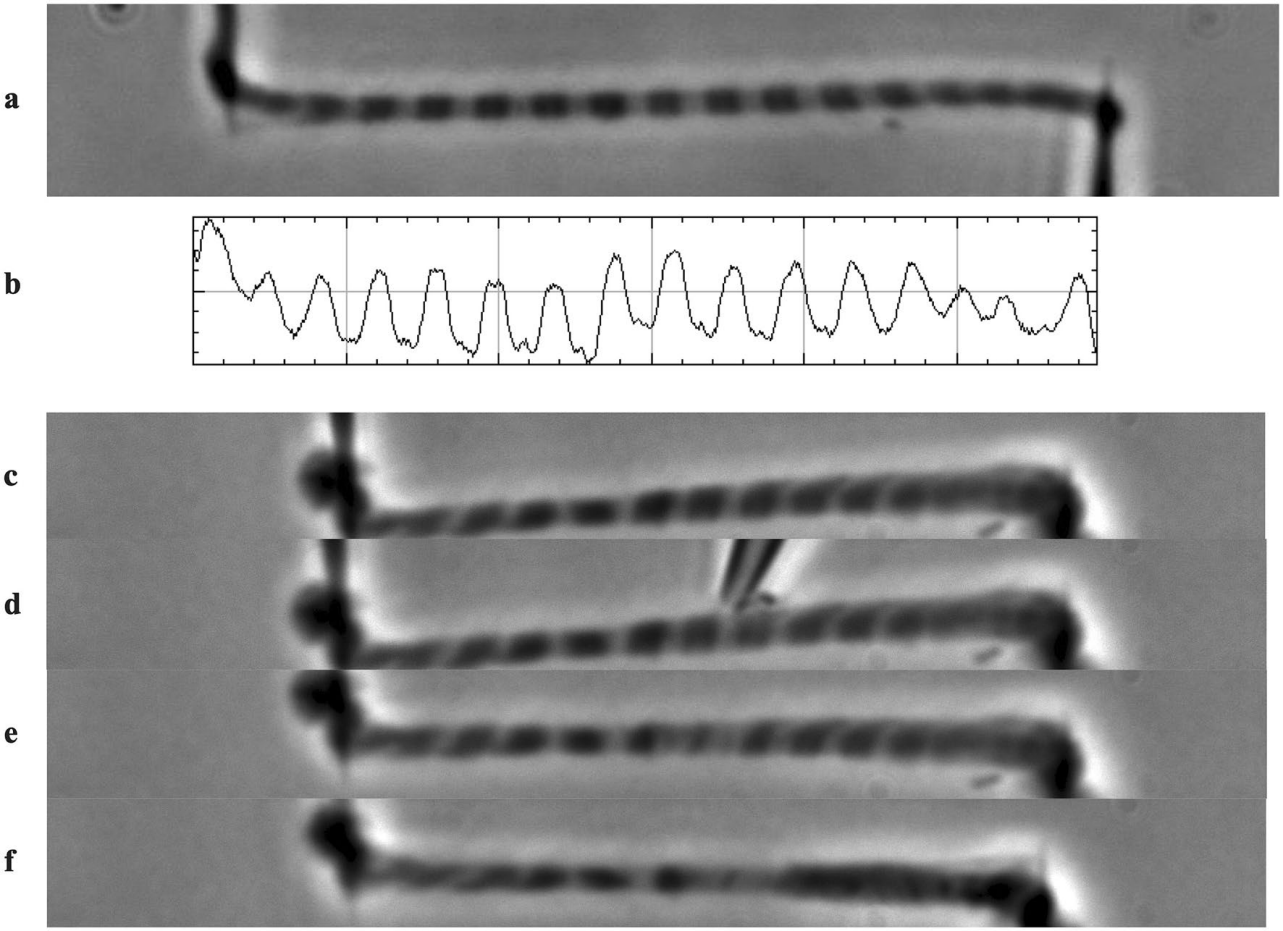
(**a**): An image of a single myofibril suspended between two glass needles and visualized at high magnification. (**b**) Plot profile matching the greyscale pattern of the myofibril in (**a**). The dark A-bands of the sarcomeres produce peaks that are used in the calculation of sarcomere lengths. (**c**) A myofibril with an average sarcomere length of 2.73 μm at rest. (**d**) The tip of the pressure-controlled micro-perfusion system filled with high ionic strength solution is visible above the myofibril. The tip is positioned such that a single sarcomere will be affected by the solution. (**e**) Contractile proteins in a single sarcomere were inactivated by applying high ionic strength solution. Note the reduced appearance of the A-band and more visible Z-lines. (**f**) Myofibril with one inactive sarcomere in a contracted state.

In this study, the contractile properties of a single myofibril were compared before and after localized treatment of one central sarcomere with a high ionic strength solution. Figure 1 shows consecutive points in time during a typical experiment. While activating and relaxing was done using a large perfusion system surrounding the whole myofibril (not pictured in the Figure), localized treatment with high ionic strength solution only affected a single sarcomere. The micro-perfusion pipette utilized for the targeted treatment of a single sarcomere was brought close to the myofibril. When external pressure was applied to the micro-perfusion, a flow of high ionic strength solution was created that only affected the selected sarcomere (Fig. 1e,f). The result is an intact myofibril in which the A-band of a single sarcomere is inactivated, hereafter referred to as a myofibril with an “inactive” sarcomere. Most importantly, in the context of this study, inactivating one sarcomere in a myofibril with several sarcomeres increased the non-uniformity of sarcomere lengths, visible in Fig. 1e,f.

### Control forces

Three isometric contractions were developed before depletion of the thick filament and were compared to ensure reproducibility of the data and to evaluate potential damage to the myofibrils during the experiments. The force values were not significantly different (*P* = 0.87). The same analysis was performed with isometric contractions developed after depletion of the thick filament, and the result was similar (*P* = 0.82). Finally, a comparison was made between the two successive stretch contractions that were developed before and also the two stretch contractions that were developed after depletion of the thick filament. The forces were not statistically different (*P* = 0.79 and *P* = 0.82, respectively) within the two groups of myofibrils (control and sarcomere-depleted group).

### Isometric forces

Force values taken during isometric contractions developed at a sarcomere length of 2.76 ± 0.02 μm in the control group averaged 103.84 ± 5.1 nN/μm^2^ (n = 11). In contrast, myofibrils with an inactive sarcomere produced an average force of 94.03 ± 6.20nN/μm^2^ at a sarcomere length of 2.77 ± 0.01 μm (n = 11). Therefore, the inactivation of a single sarcomere caused a force decrease of 9.92 ± 3.17%. These values are within the range of those obtained in previous studies using similar experimental protocols in our laboratories^8,18^.

**Figure 2:**
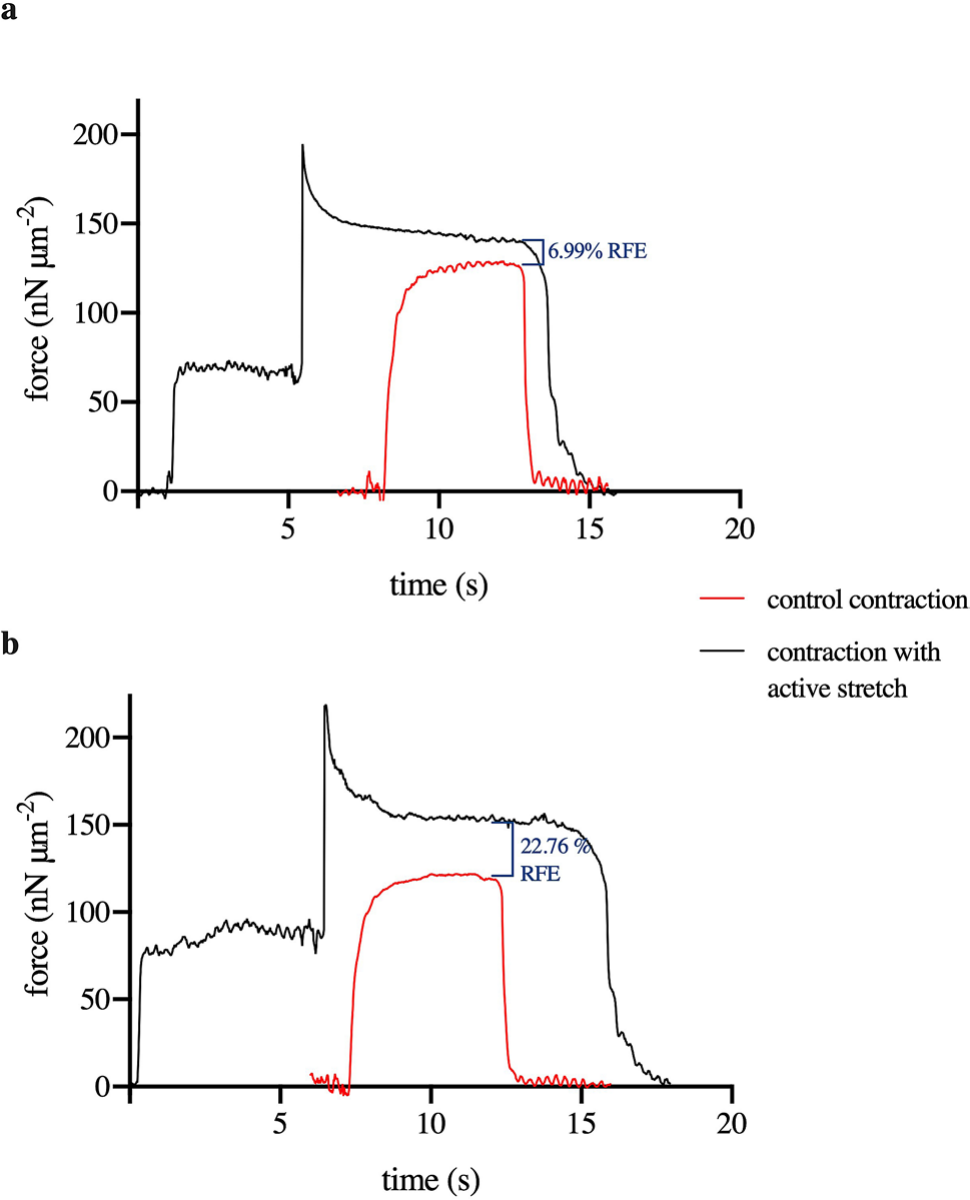
Contractions of a control myofibril (**a**) and a myofibril treated with high ionic strength solution (**b**). The red traces show an isometric contraction, while the black traces show contractions with an active stretch. Residual force enhancement was calculated as the difference between the force following the stretch and the isometric force. In this myofibril, the residual force enhancement was 6.99% and 22.76% for control and treated myofibril, respectively. Forces were measured at the same sarcomere length, which in this case, was 2.73 μm.

Figure 2 shows typical force traces of a control myofibril (a) and a myofibril with one inactive sarcomere (b). The isometric force of the control myofibril in this example showed a total active force of 129.85 nN/μm^2^, while the myofibril with one inactive sarcomere produced a total active force of 122.29 nN/μm^2^.

### Residual force enhancement

To measure the residual force enhancement, isometric forces and forces following a stretch step were compared at similar sarcomere lengths. The top graph in Fig. 2 shows traces of an intact myofibril contracting isometrically (red) and then contracting with an active stretch step induced during full force development (black). The isometric contraction of this myofibril (red) was developed at a sarcomere length of 2.73 μm. The black-traced contraction was started at a shorter sarcomere length which was reduced by 20%. Subsequently, the myofibril was activated and stretched to a sarcomere length similar to that of the isometric contraction (2.73 μm) during the ongoing activation. In this myofibril, the isometric contraction produced a force of 129.85nN/μm^2^ while the contraction following stretch produced a force of 138.94 nN/μm^2^. Hence, there was an increase in force of 9.1 nN/μm^2^ or 7.01%.

**Figure 3:**
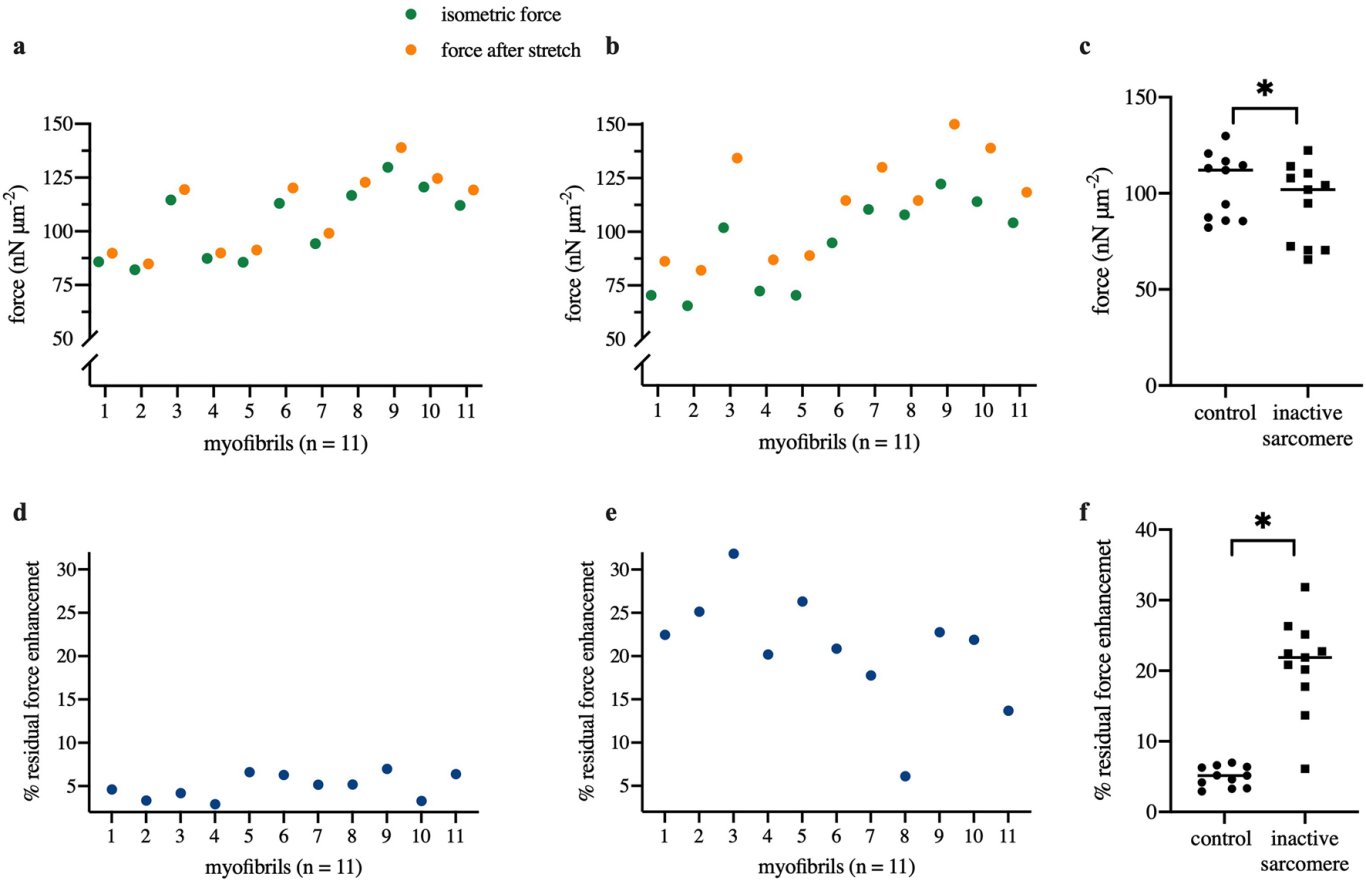
Force values of control (**a**) and treated (**b**) myofibrils during isometric contractions (green) and after active stretching (yellow). (**c**) Isometric forces were significantly lower in myofibrils with an inactive sarcomere compared to control myofibrils (*P* < 0.01). Residual force enhancement was observed in both control myofibrils (**d**) and myofibrils with one inactive sarcomere (**e**). (**f**) The percent residual force enhancement at a comparable sarcomere length was significantly larger in myofibrils with an inactive sarcomere compared to control myofibrils (*P* < 0.01).

The force enhancement was also observed after one sarcomere in the same myofibril was inactivated (Fig. 2b). In this case, the isometric force decreased to 122.29 nN/μm^2^ after depletion of the thick filaments, but the residual force enhancement following stretch was larger than that observed in the untreated myofibril, with a force of 150.13 nN/μm^2^—an increase of 27.84 nN/μm^2^ or 22.77%. A summary of all forces measured during the experiments is presented in Fig. 3 (panel (a): control myofibrils, panel (b): myofibrils with one inactive sarcomere), and for better comparison depicted in one graph in panel (c). The residual force enhancement is shown in Fig. 3 (panels (d,e)), for control myofibrils and myofibrils with one inactive sarcomere, respectively. Our results show a residual force enhancement ranging from 5.00 ± 0.44% in control myofibrils to 20.82 ± 2.03% in treated myofibrils. Therefore, the inactivation of one sarcomere within the myofibrils tested lead to a significant increase in the residual force enhancement (Fig. 3f).

### Sarcomere length dispersion

**Figure 4:**
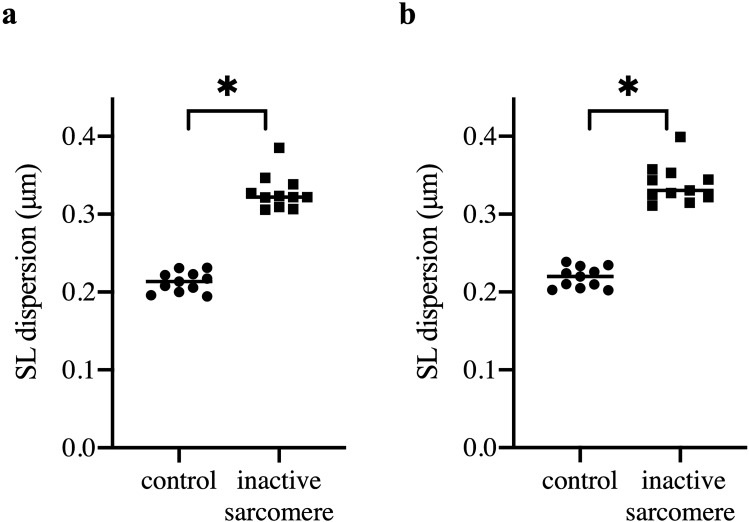
Sarcomere length (SL) dispersion of the myofibrils. The inactivation of a single sarcomere within the myofibril caused a significant increase in sarcomere length dispersion both before (**a**) and during (**b**) contraction (*P* < 0.01).

To investigate the connection between sarcomere length non-uniformity and residual force enhancement, sarcomere length dispersion was calculated in each myofibril. Figure 4 shows that sarcomere length dispersion increased during activation in both groups of myofibrils. Control myofibrils showed an average sarcomere length dispersion of 0.21 ± 0.004 μm at rest (Fig. 4a), and the dispersion increased slightly during the contraction to 0.22 ± 0.004 μm (Fig. 4b). Sarcomere length dispersion was significantly higher once one sarcomere was inactivated (*P* < 0.01) with values of 0.33 ± 0.007 μm and 0.34 ± 0.008 μm for relaxed and contracted states (Fig. 4a,b), respectively. These values are similar to results from previous studies^19,20^.

## Discussion

In this study, we investigated the residual force enhancement in skeletal muscles and its connection to sarcomere-length non-uniformities. A novel technique developed in our laboratory allowed us to introduce targeted sarcomere non-uniformity in a myofibril and directly compare its effect on force production before and after stretch. Our main findings were: (i) residual force enhancement after stretch was observed in all myofibrils tested, (ii) the inactivation of a selected sarcomere increased both sarcomere length dispersion and the level of residual force enhancement, and (iii) there was a close relationship between the increase in sarcomere length dispersion and the residual force enhancement. Force values obtained in the current study are similar to those obtained previously in experiments with myofibrils^16,17,21^, and the level of residual force enhancement is within the range of values in the literature^8,11,15^.

### Comparison to other studies

Residual force enhancement has been a popular subject of investigation, because it cannot readily be explained by the classic isometric force-sarcomere length relation^1^. In our experiments, the force developed during an isometric contraction was first allowed to stabilize, upon which a stretch of 20% relative to the starting length of the myofibril was imposed. The force obtained after stretch was, on average, 5% greater than the isometric force developed at the same sarcomere length in control myofibrils, a force increase that is comparable to past studies^8,11,15^. Although there is a scarcity of studies that carefully compared forces produced by myofibrils and sarcomeres during contractions while controlling for sarcomere length and sample integrity, studies that use a rigorous experimental protocol report an average force enhancement of 5–40% in single fibers^5,6,22^, myofibrils^8,11,15^, and single sarcomeres^8,21^.

### Mechanism

One of the main theories explaining the underlying mechanism of residual force enhancement is the occurrence of sarcomere length non-uniformities that arise upon contraction of a myofibril^6,13,14^. When activated, sarcomeres at different lengths elicit different responses—shorter, stronger sarcomeres will contract at the expense of longer, weaker sarcomeres that will stretch^3,6^. Imposing a stretch during an active contraction exaggerates these irregularities in sarcomere lengths; strong sarcomeres will have a favourable overlap, producing higher active forces, while weak sarcomeres elongate to greater lengths shifting their contribution to the overall force output. This scenario allows for an increase in the total force: shorter sarcomeres have a larger number of myosin heads attached to actin, producing more active force. The sarcomeres that will be stretched lose filament overlap but will have a greater stiffness due to the elongation of titin, which acts as a spring-like molecule and is responsible for passive forces in sarcomeres. The overall force produced by both the shorter sarcomeres with more cross-bridge interaction and the longer sarcomeres with a large passive force will be higher than the force produced during isometric contractions.

To test the hypothesis that sarcomere length non-uniformities contribute to residual force enhancement, we utilized a novel method that induces targeted non-uniformity by inactivation of the contractile proteins in a single sarcomere. We observed a decline in isometric force with the inactivation of contractile proteins in one sarcomere, similar to findings from studies using the same experimental method that found a 4% to 30% decrease in forces with the extraction of consecutive sarcomeres^16,18^. In this study, we observed a ~ 10% force decline in myofibrils with one inactive sarcomere compared to control myofibrils at the same sarcomere length. These results suggest that the total force of a myofibril is not only related to the cross-sectional area and length of a myofibril but also to the number of active sarcomeres in series. Furthermore, our results show that inactivating one sarcomere effectively increases non-uniformity and the sustained force after stretch is ~ 20% higher in these myofibrils compared to the isometric force produced at the same sarcomere length. While control myofibrils produce a residual force enhancement of ~ 5%, myofibrils with one inactive sarcomere show four times larger levels of residual force enhancement. The increase in residual force enhancement observed in this study is in agreement with a study that reported a linear correlation between the degree of half-sarcomere non-uniformity and the magnitude of force enhancement after stretch in myofibrils^8^.

Some myofibrils in the current study showed a negligible amount of sarcomere length non-uniformity, and yet still produced an enhanced force after stretch. These results suggest that non-uniformity is, in part, connected to residual force enhancement, but that it cannot account for the phenomenon in its entirety^7,8,15^. Residual force enhancement has been observed at shorter sarcomere lengths, along the ascending limb of the force–length relation, where sarcomere non-uniformities are minimal^15^, suggesting that the mechanism of residual force enhancement has components independent of sarcomere inhomogeneity. The occurrence of residual force enhancement in single sarcomeres—preparations in which non-uniformity is naturally absent—also suggests an alternate mechanism for enhanced forces after stretch^21^.

A possible explanation is centered around the Ca^2+^-dependent increase in stiffness of the elastic protein titin. When Ca^2+^ levels rise during activation of myofibrils, Ca^2+^ binds to the PEVK-domains of titin, which consequently reduces its persistence length^23^. This leads to an increase in the stiffness of titin and a subsequently higher contribution of passive forces to the total force output after an imposed stretch^24^. When a myofibril is stretched, the stiffness of titin will increase further causing a larger level of passive force contribution to the total force. This mechanism has been investigated in recent studies that linked levels of a static tension, that cannot be accounted for myosin-actin interactions, to the amount of residual force enhancement seen in myofibrils^22^ and muscle fibers^12^. Interestingly, myofibrils isolated from muscles containing different isoforms of titin present different responses to stretch. Studies specifically designed to compare different muscle types showed that myofibrils isolated from the soleus and psoas show residual force enhancement that is accompanied by an increase in the static stiffness, while cardiac myofibrils do not show the residual force enhancement^11,22^. The difference may be related to the physiological role of titin. Cardiac muscles are not stretched while contracting in vivo, and thus a residual force enhancement caused by a Ca^2+^-dependent increase in titin stiffness would not play a physiological role during contractions. This interpretation is strengthened by a recent study showing that intact cardiac trabeculae also do not present the residual force enhancement after stretch^25^.

## Conclusion

Sarcomere length non-uniformity is partially responsible for the residual force enhancement after stretch of skeletal muscle myofibrils.

## Methods

### Sample preparation

Rabbit psoas samples were obtained and stored as described previously^26^. The procedure was in accordance with the McGill University Animal Care Committee and the Canadian Council on Animal Care, and was approved by protocol #5227 by the Facility Animal Care Committee (FACC). Shortly, samples were stored in a 50:50 rigor:glycerol solution and defrosted in rigor solution at 4 °C for 1 h before mechanical experimentation. The muscle sample was then homogenized in six consecutive steps (2 × 8000 rpm, 2 × 15,000 rpm, 2 × 21,000 rpm) with a VWR 250 homogenizer. The homogenate was transferred to a temperature-controlled experimental chamber (10 °C) and mounted on a phase-contrast microscope. The sample was washed three times with rigor solution and finally immersed in relaxing solution.

### Solutions

Three solutions were used for sample preparation and experimentation: rigor solution, relaxing solution and activating solution. The rigor solution was composed of (in mM): 50 Tris, 100 KCl, 2 MgCl_2_, and 1 EGTA (pH 7.0). The relaxing solution was composed of (in mM); 7 EGTA, 20 Imidazole, 5 MgCl_2_, 69 KCl, 6 ATP and 19 CrP (pH 7.0) and the activation solution was composed of (in mM): 7 CaCl_2_, 7 EGTA, 20 imidazole, 5 MgCl_2_, 52 KCl, 6 ATP and 19 Crp (pH 7.0). Furthermore, a high ionic strength solution was used to inactivate acto-myosin interaction in individual sarcomeres and was composed of relaxing solution and 800 mM KCl.

### Experimental setup

Two glass micro-needles were used for the fixation of selected myofibrils during the experiments. The needles were produced with a pipette puller (KOPF 720; David Kopf Inst.), and the stiffness of the needles was calculated by the cross bending method^27^ using a cantilever with a stiffness of 34.89 nN/μm. The resulting stiffnesses of the glass needles used in this study ranged from 30.6nN/μm to 56.9 nN/μm. Both needles were controlled by micro-manipulators (NT88-V-Nikon). One of the needles was connected to a computer-controlled piezo motor to induce needle movement (stretch steps) during contractions. The micro-perfusion system used for additional treatment of selected sarcomeres was produced with the same needle puller using a hollow glass capillary. The tip of the micro-perfusion was adjusted to 1 μm using a microforge (MF-900; Narishige) and filled with a high ionic strength solution.

Contractions of myofibrils were induced by a fast switching system that allowed exchanges between relaxing and activating solutions delivered from a double-barrelled perfusion pipette, that was directed at the selected myofibril, as described in details elsewhere^8^.

The surface of the experimental chamber was visualized by an inverted phase-contrast microscope (Eclipse TE2000-U) under high magnification (Nikon Plan Fluor, X100, numerical aperture 1.30 + 1.5 × microscope magnification). All experiments were video-recorded (Hamamatsu Orca-ER digital camera) for subsequent data analysis.

### Experimental protocol

A clear striation pattern (dark A-bands and light I-band) was used to select myofibrils for mechanical experimentation once they were in the experimental chamber. Myofibrils were pierced parallel to the Z-lines by the two pre-calibrated glass micro-needles and lifted from the surface of the chamber. The myofibrils were adjusted so that the nominal average sarcomere length was 2.8 μm (actual: 2.76 ± 0.02 μm, as verified post-analysis) before a contraction was induced by the activation solution. After fast activation, contractions were held steady for 15 s before the surrounding solution was switched to the relaxing solution, which induced full relaxation of the myofibril. After two control contractions, the sarcomere length was reduced by 20%. During the subsequent activation of the myofibril, a stretch of 20% of the total length at a speed of 0.3 μm/s was induced during force production to reach a sarcomere length similar to the purely isometric contraction (actual: 2.77 ± 0.01 μm). The force was then allowed to reach a steady-state before relaxation. With this method of matching sarcomere lengths through a stretch step, force production could be compared in the different contractions, isometric and post-stretch, at similar average sarcomere lengths. The stretch was repeated twice to ensure reproducibility. After stretch, an isometric contraction was repeated again to check for potential damage of the myofibril.

Next, myofibrils were treated with a high ionic strength solution administered by the micro-perfusion system targeting one select sarcomere to deplete the A-band, a procedure described in detail elsewhere^16^. Briefly, a glass micropipette filled with high ionic strength (HIS) solution and a diameter at the opening of ~ 1 μm, was used to treat a single sarcomere, while a steady stream of relaxing solution was flowing through the whole myofibril to ensure a similar laminal flow. The micro-perfusion was then removed from the experimental chamber to avoid any additional sarcomeres to be affected. The high ionic solution denatures the thick filaments^28^, and therefore acto-myosin interactions were inhibited in the targeted sarcomere. Subsequent contractions were induced as described in the preceding paragraph after myofibrils were treated with high ionic strength solution (isometric contractions and contractions superimposed by a stretch). The isometric and post-stretch force values were compared at average sarcomere lengths of 2.74 ± 0.01 μm and 2.71 ± 0.04 μm, before and after thick filament depletion, respectively.

### Data analysis

The force produced by myofibrils was calculated by tracking the absolute needle displacement using the video images during the contraction. Knowing the stiffness of each needle (K) as well as the displacement of the needles (Δd), the total force (F) was calculated as:

$$F= \Delta d \left(\frac{{K}_{1}{K}_{2}}{{K}_{1}+{K}_{2}}\right)$$

Needle tracing was done with Fiji and Image J, and the integrated TrackMate plug-in. Force values were normalized by the myofibril cross-sectional area, which was calculated as an average of three diameter measures made across the myofibril. Only myofibrils that produced similar forces during the first contraction and a control contraction after the stretch protocol were included in the data set to ensure that myofibrils did not lose contractile ability after contractions and stretches, i.e., all myofibrils included in data analysis showed less than a 7% difference between the first and the last contraction.

### Residual force enhancement

Residual force enhancement was calculated as the difference between the steady state force after stretch and the isometric force at a similar sarcomere length. Both force values were measured as an average calculated over a 5 s period when force has stabilized after the initial rise in isometric contractions or after stretch.

### Sarcomere length measurements

Two measures of sarcomere length were taken for each myofibril: the average sarcomere length and the individual sarcomere lengths. The average sarcomere length was taken as the total length of a given myofibril divided by the number of sarcomeres in series. The individual sarcomere length was measured using a greyscale plot, as depicted in Fig. 1B, using the Fiji software. Z-lines were identified, and the distance between Z-lines was taken as a measure of individual sarcomere length^18^. Sarcomere dispersion was calculated as the absolute difference between the individual sarcomere length and the average sarcomere length for each myofibril and was used as a measure for sarcomere length non-uniformity.

$${SL}_{dispersion}=\left|{SL}_{average}-{SL}_{individual}\right|$$

### Statistical analysis

All data collected during the experiments was normally distributed. A one-way analysis of variance (ANOVA) for repeated measures was performed to compare the three isometric contractions developed before depletion of thick filaments, and also to compare the three isometric contractions developed after depletion of the thick filaments. These comparisons were made to ensure reproducibility of the data and check for potential damage to the myofibrils during the experiments. The same comparison was made between the two successive stretch contractions developed before, and also the two contractions developed after depletion of the thick filament. A two-way ANOVA for repeated measures was used to identify potential differences in forces developed by myofibrils during control and post-stretch contractions, before and after extraction of the thick filaments. A level of significance of *P* ≤ 0.05 was set for all comparisons. All results are presented as means ± standard error (SEM).

All analyses were done in using GraphPad Prism version 9.0.0 for macOS.
